# Supplementary material for: Medication abortion via digital health in the United States: a systematic scoping review
Source: NPJ Digit Med. 2023 Jul 12;6:128. doi: 10.1038/s41746-023-00871-2 (PMC10338479; doi:10.1038/s41746-023-00871-2)
Supplement: Supplementary file 1 — Reporting Summary [file 41746_2023_871_MOESM1_ESM.pdf]

nature portfolio

Corresponding author(s): Fekede Kumsa & Arash Shaban-Nejad

Last updated by author(s): May 5, 2023

Reporting Summary

Nature Portfolio wishes to improve the reproducibility of the work that we publish. This form provides structure for consistency and transparency in reporting. For further information on Nature Portfolio policies, see our Editorial Policies and the Editorial Policy Checklist.

Statistics

For all statistical analyses, confirm that the following items are present in the figure legend, table legend, main text, or Methods section.

n/a	Confirmed	
☒	☐	The exact sample size (*n*) for each experimental group/condition, given as a discrete number and unit of measurement
☒	☐	A statement on whether measurements were taken from distinct samples or whether the same sample was measured repeatedly
☒	☐	The statistical test(s) used AND whether they are one- or two-sided<br>*Only common tests should be described solely by name; describe more complex techniques in the Methods section.*
☒	☐	A description of all covariates tested
☒	☐	A description of any assumptions or corrections, such as tests of normality and adjustment for multiple comparisons
☒	☐	A full description of the statistical parameters including central tendency (e.g. means) or other basic estimates (e.g. regression coefficient) AND variation (e.g. standard deviation) or associated estimates of uncertainty (e.g. confidence intervals)
☒	☐	For null hypothesis testing, the test statistic (e.g. *F*, *t*, *r*) with confidence intervals, effect sizes, degrees of freedom and *P* value noted<br>*Give P values as exact values whenever suitable.*
☒	☐	For Bayesian analysis, information on the choice of priors and Markov chain Monte Carlo settings
☒	☐	For hierarchical and complex designs, identification of the appropriate level for tests and full reporting of outcomes
☒	☐	Estimates of effect sizes (e.g. Cohen's *d*, Pearson's *r*), indicating how they were calculated

Our web collection on statistics for biologists contains articles on many of the points above.

Software and code

Policy information about availability of computer code

Data collection	Not applicable
Data analysis	Not applicable

For manuscripts utilizing custom algorithms or software that are central to the research but not yet described in published literature, software must be made available to editors and reviewers. We strongly encourage code deposition in a community repository (e.g. GitHub). See the Nature Portfolio guidelines for submitting code & software for further information.

Data

Policy information about availability of data

All manuscripts must include a data availability statement. This statement should provide the following information, where applicable:

- Accession codes, unique identifiers, or web links for publicly available datasets
- A description of any restrictions on data availability
- For clinical datasets or third party data, please ensure that the statement adheres to our policy

This study is scoping review from the publicly available studies and all extracted data are included in the review.

Research involving human participants, their data, or biological material

Policy information about studies with human participants or human data. See also policy information about sex, gender (identity/presentation), and sexual orientation and race, ethnicity and racism.

Reporting on sex and gender	We used gender inclusive terms throughout our reporting. The nature of our study doest not necessitate subgroup analysis based on sex and gender.
Reporting on race, ethnicity, or other socially relevant groupings	We did not make any reporting based on race, ethnicity, or other socially relevant groupings since our synthesis did not let us to do so.
Population characteristics	We only performed narrative synthesis of medication abortion services utilization via telemedicine. We did not extracted and used any individual participants characteristics. However, characteristics of the included studies were clearly described in the study. Of the 33 studies included in the final review, 11 were qualitative studies, six were cross-sectional studies, and were cohort (prospective, retrospective, or follow-up) studies. One study used a mixed methods approach, one used a multicenter single-arm clinical trial, and one used an investigational new drug application (INDA) approach. The participants in 26 studies were potential abortion users, the participants of two studies included both abortion users and providers, and those of the remaining five studies were abortion care providers.
Recruitment	Not applicable
Ethics oversight	Not applicable

Note that full information on the approval of the study protocol must also be provided in the manuscript.

Field-specific reporting

Please select the one below that is the best fit for your research. If you are not sure, read the appropriate sections before making your selection.

☒ Life sciences ☐ Behavioural & social sciences ☐ Ecological, evolutionary & environmental sciences

For a reference copy of the document with all sections, see nature.com/documents/nr-reporting-summary-flat.pdf

Life sciences study design

All studies must disclose on these points even when the disclosure is negative.

Sample size	Given this study is scoping review, we did not employ any statistical analysis. However, 33 studies consisting 128,378 participants included.
Data exclusions	We excluded studies that were ineligible on the basis of our exclusion criteria, including duplicate studies, conference papers, anonymous reports, and review papers, and did not report any finding related to medication abortion via telemedicine, editorials, and theoretical papers.
Replication	Not applicable
Randomization	Not applicable
Blinding	Not applicable

Reporting for specific materials, systems and methods

We require information from authors about some types of materials, experimental systems and methods used in many studies. Here, indicate whether each material, system or method listed is relevant to your study. If you are not sure if a list item applies to your research, read the appropriate section before selecting a response.

Materials & experimental systems

n/a	Involved in the study	
☒	☐	Antibodies
☒	☐	Eukaryotic cell lines
☒	☐	Palaeontology and archaeology
☒	☐	Animals and other organisms
☒	☐	Clinical data
☒	☐	Dual use research of concern
☒	☐	Plants

Methods

n/a	Involved in the study	
☒	☐	ChIP-seq
☒	☐	Flow cytometry
☒	☐	MRI-based neuroimaging
